# Association of dietary index for gut microbiota and cardiovascular diseases in American adults: evidence from National Health and Nutrition Examination Survey 1999–2018

**DOI:** 10.3389/fnut.2025.1604891

**Published:** 2025-07-02

**Authors:** Hong Luo, Wujie Xia, Liya Pan

**Affiliations:** Department of Cardiology, The Second Affiliated Hospital and Yuying Children's Hospital of Wenzhou Medical University, Wenzhou, China

**Keywords:** DI-GM, cardiovascular disease, NHANES, diet index, gut microbiota

## Abstract

**Background:**

As a significant health burden, cardiovascular disease (CVD) contributes substantially to global disease and death rates. While the gut microbiota has been linked to CVD, its dietary influence remains unclear. The Dietary index for gut microbiota (DI-GM) evaluates diet-related impacts on microbiota diversity. This study explores the relationship between DI-GM and the risk of CVD.

**Methods:**

Using National Health and Nutrition Examination Survey (NHANES) data spanning 1999–2018, this cross-sectional study included 39,181 adults aged 20 years or older, among whom were individuals with CVD. DI-GM, based on dietary recall, was calculated to assess microbiota diversity. Multivariable weighted logistic regression explored the association between DI-GM and CVD, with trend tests, subgroup analyses, smoothed curves, and multiple imputation ensuring robustness. Mediation analysis examined the role of body mass index (BMI).

**Results:**

A total of 39,181 participants (mean age: 47.15 years) were included, with 49.18% female and 50.82% male. Higher DI-GM levels correlated with a decreased prevalence of CVD (DI-GM: OR = 0.95, 95% CI = 0.92–0.98). Compared to participants with DI-GM scores of 0–3, those with DI-GM ≥6 had significantly lower CVD risk (OR = 0.82, 95% CI = 0.71–0.94). Restricted cubic spline analysis showed a linear association between DI-GM and CVD. A significant mediating effect of BMI was observed (proportion of mediation: 16.27%, 95% CI: 9.11%−35.48%).

**Conclusions:**

An inverse association was found between the DI-GM index and CVD prevalence, where increased DI-GM scores corresponded to a lower CVD risk, partly mediated by reductions in BMI.

## Introduction

Cardiovascular disease (CVD) is the leading cause of death globally (1). responsible for approximately 18 million deaths annually—accounting for over 30% of all global mortality, according to the World Health Organization (2, 3). While hypertension, smoking, and metabolic disorders are established risk factors (4–7), growing evidence highlights the important role of dietary patterns in shaping cardiovascular health. Among emerging mechanisms, the gut microbiota has gained attention in recent research, with findings showing its role in modulating metabolism, inflammation, and vascular function (8–10).

Diet is a primary modulator of gut microbiota composition and diversity, and may influence cardiovascular outcomes via microbial metabolites and inflammatory pathways. In particular, short-chain fatty acids (SCFAs) and trimethylamine N-oxide (TMAO), generated through microbial fermentation, have been linked to blood pressure regulation, lipid metabolism, and systemic inflammation—processes central to CVD development (11). Building on this diet–microbiota–CVD axis, Kase et al. reviewed studies on diet-microbiota interactions and identified 14 dietary components that influence gut microbiota (12). This led to the creation of the Dietary Index for Gut Microbiota (DI-GM), a tool that evaluates diet quality based on its impact on gut microbiota diversity. DI-GM includes 10 beneficial components (e.g., fiber, fermented dairy, and plant-based foods) and four harmful components (e.g., red meat and processed foods). Although DI-GM has been associated with conditions such as depression, constipation, and diabetes (13–15), its relationship with CVD remains underexplored.

Despite increasing interest in the gut microbiota's role in cardiometabolic health, no study has specifically examined the association between the DI-GM and CVD in a nationally representative population. Therefore, this study aims to investigate the association between DI-GM and CVD among U.S. adults using NHANES data, and to explore the potential mediating role of body mass index (BMI). Our findings may offer novel insights into the interplay between diet quality, gut microbiota, and cardiovascular risk.

## Materials and methods

### Data source

This study utilized data from the National Health and Nutrition Examination Survey (NHANES), which was conducted from 1999 to 2018. NHANES, managed by the National Center for Health Statistics (NCHS), is an ongoing, cross-sectional survey aimed at assessing the health and nutritional status of the non-institutionalized U.S. population (16). The survey uses a complex, multistage probability sampling design involving stratification and clustering to ensure national representativeness. Data collection includes structured household interviews, standardized physical examinations in mobile examination centers (MECs), and laboratory tests. Approved by the NCHS Ethics Review Board, the data are publicly available, with written consent obtained from all participants. The survey follows the Strengthening the Reporting of Observational Studies in Epidemiology (STROBE) guidelines. Additional details about NHANES can be found at https://www.cdc.gov/nchs/nhanes.

### Study population

**Figure 1:**
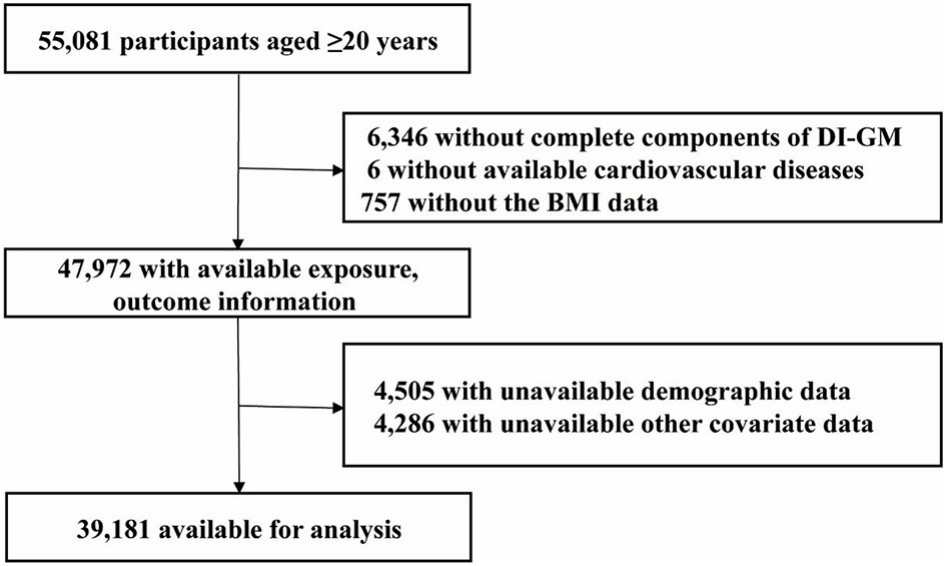
The study's flow diagram. DI-GM, dietary index for gut microbiota; BMI, body mass index.

Our analysis used NHANES data from 1999 to 2018, focusing on participants aged 20 years and older (*n* = 55,081). Exclusion criteria were based on missing data in the following variables: components of the DI-GM index (*n* = 6,346), cardiovascular disease (CVD; *n* = 6), BMI (*n* = 757), demographic information (*n* = 4,505), and data on smoking status, physical activity, alcohol intake, hypertension, hyperlipidemia, and diabetes mellitus (*n* = 4,286). After applying these exclusions, 39,181 participants were considered in the final analysis (Figure 1).

### Definitions of CVD

CVD was defined based on self-reported physician diagnoses using standardized questionnaires administered by trained interviewers during the NHANES household interview. Participants were asked whether a doctor or other health professional had ever told them that they had any of the following conditions: (1) congestive heart failure, (2) coronary heart disease, (3) angina (angina pectoris), (4) heart attack (myocardial infarction), or (5) stroke. An affirmative response to any of these five questions was used to classify the participant as having CVD. This definition is consistent with previous studies using NHANES data to assess CVD prevalence and risk.

### Measurement of dietary index for gut microbiota

The DI-GM serves as a dietary quality index aimed at assessing how dietary patterns influence gut microbiota diversity. It comprises 14 food items or nutrients, classified as either beneficial or unfavorable for gut health. Beneficial components, which promote a healthy microbiome, include avocado, fiber, broccoli, cranberries, chickpeas, fermented dairy, coffee, soy, and whole grains, along with green tea (though NHANES did not specifically record green tea consumption). Unfavorable components, which may harm microbiota diversity, include red and processed meats, refined grains, and diets high in fat (≥40% of total energy from fat) (12). Dietary data for DI-GM calculation were obtained through the USDA's 24-h recall method (Automated Multiple-Pass Method, AMPM), conducted by trained interviewers. This method records all foods and beverages consumed by participants in the previous 24 h, with standardized protocols to minimize bias.

For scoring, beneficial components received a score of 1 if intake was greater than the sex-specific median, and 0 if below. For unfavorable components, a score of 1 was assigned if consumption was below the median (or if fat intake was < 40% of total energy), and 0 if above. The individual scores were combined to generate the overall DI-GM score, which ranges from 0 to 13. The scores are categorized into four groups: 0–3, 4, 5, and ≥6, with higher scores indicating more favorable dietary patterns for gut health. Comprehensive details regarding the components and scoring criteria can be found in Supplementary Table S1.

### Definitions of BMI

BMI was determined by dividing weight (kg) by height squared (m^2^).

### Assessment of covariates

Covariate data were gathered using baseline questionnaires completed by trained professionals. Participants provided details on age, gender, race, marital status, education and poverty-to-income ratio (PIR). Smoking status was classified as current, former, or never, and drinking status was grouped as never, former, light, moderate, or heavy. Physical activity was quantified based on the Metabolic Equivalent of Task (MET). Hypertension criteria included systolic blood pressure ≥140 mmHg, diastolic ≥90 mmHg, physician diagnosis, or ongoing use of antihypertensive drugs. Hyperlipidemia was defined by hypertriglyceridemia (≥150 mg/dl), abnormal cholesterol levels, or use of lipid-lowering medications. Diabetes was determined by diagnosis, fasting glucose ≥7.0 mmol/L, HbA1c ≥6.5%, or the use of diabetes drugs.

### Statistical analysis

Due to the complex multistage cluster survey design of NHANES, we chose the dietary day one weights from NHANES (1999–2018) as our sample weights, using the codes “WTDR4YR” (1999–2002) and “WTDRD1” (2003–2018). Specifically, for 1999–2002, weights were calculated as 2/10 × WTDR4YR, and for 2003–2018, as 1/10 × WTDRD1.

Participant characteristics were stratified by the presence or absence of cardiovascular disease (CVD). Categorical variables were reported as unweighted counts with weighted proportions, while continuous variables were expressed as means with standard errors (SE). The Wilcoxon rank-sum test was used for continuous variables in complex survey data, and the Rao-Scott chi-square test was applied for categorical variables. Weighted multivariable logistic regression estimated the association between DI-GM and CVD, providing odds ratios (OR) with 95% confidence intervals (CIs). Crude models were not adjusted for potential confounders. Model 1 accounted for age and gender, while Model 2 additionally controlled for race, marital status, education level, and PIR. Model 3 further adjusted for smoking and drinking status, physical activity, hypertension, diabetes, hyperlipidemia, and energy intake. DI-GM was categorized into four groups (0–3, 4, 5, ≥6) to examine the effect of DI-GM on CVD risk. To explore potential nonlinear relationships, a restricted cubic spline model was used, adjusting for variables in Model 3. Subgroup analyses were performed by categorizing participants based on age, gender, smoking status, hypertension, diabetes, and hyperlipidemia, with adjustments following Model 3. Sensitivity analyses excluded participants with extreme energy intake (< 500 or >5,000 kcal per day).

All analyses were performed using R 4.2.2 (R Foundation) and Free Statistics 2.1, with statistical significance set at *P* < 0.05.

## Results

### Baseline characteristics of study subjects

Table 1 presents the baseline characteristics of the study subjects, representing 182.46 million U.S. adults with a mean age of 47.15 years (SE, 0.21). Among them, 16.14 million individuals were identified with CVD. Compared to those without CVD, individuals with CVD were significantly older, more likely to be male, have lower levels of physical activity, higher BMI, and a higher prevalence of hypertension, diabetes, and hyperlipidemia. Additionally, the CVD group exhibited less favorable dietary patterns in terms of gut microbiota, as indicated by lower scores for beneficial dietary components and higher scores for unfavorable dietary components.

**Table 1:** Characteristics of the NHANES 1999–2018 participants.

**Characteristics**	**Total**	**Without CVD**	**CVD**	***P*-value**
	***N*** = **182,456,881**	***n*** = **166,315,366**	***n*** = **16,141,515**	
Age, years	47.15 (0.21)	45.50 (0.20)	64.19 (0.32)	< 0.001
**Gender (%)**				
Male	19,703 (49.18)	17,172 (48.68)	2,531 (54.35)	< 0.001
Female	19,478 (50.82)	17,628 (51.32)	1,850 (45.65)	
**Race (%)**				
Non-Hispanic White	18,376 (70.52)	15,838 (69.91)	2,538 (76.83)	< 0.001
Non-Hispanic Black	8,020 (10.54)	7,125 (10.49)	895 (11.04)	
Mexican American	6,575 (7.68)	6,093 (8.02)	482 (4.14)	
Other Hispanic	3,024 (4.94)	2,763 (5.11)	261 (3.24)	
Other race	3,186 (6.32)	2,981 (6.48)	205 (4.75)	
**Education level (%)**				
Less than 9th grade	4,235 (5.08)	3,523 (4.63)	712 (9.77)	< 0.001
9–11th grade	5,647 (10.75)	4,858 (10.26)	789 (15.80)	
High School or equivalent	9,133 (24.10)	8,032 (23.73)	1,101 (27.93)	
Some college	11,409 (31.69)	10,253 (32.01)	1,156 (28.31)	
College graduate or above	8,757 (28.38)	8,134 (29.37)	623 (18.19)	
**Marital status (%)**				
Married or living with a partner	23,666 (62.91)	21,165 (63.17)	2,501 (60.31)	0.008
Living alone	15,515 (37.09)	13,635 (36.83)	1,880 (39.69)	
**PIR (%)**				
≤ 1.30	11,729 (21.26)	10,134 (20.50)	1,595 (29.08)	< 0.001
1.31–3.50	14,955 (35.23)	13,161 (34.75)	1,794 (40.18)	
>3.50	12,497 (43.51)	11,505 (44.75)	992 (30.74)	
**Smoking status (%)**				
Current	20,872 (53.16)	19,196 (54.67)	1,676 (37.66)	< 0.001
Former	9,948 (25.13)	8,133 (23.70)	1,815 (39.86)	
Never	8,361 (21.71)	7,471 (21.63)	890 (22.48)	
**Drinking status (%)**				
Never	5,387 (11.03)	4,761 (10.85)	626 (12.81)	< 0.001
Former	6,902 (14.31)	5,457 (12.84)	1,445 (29.54)	
Mild	13,131 (35.81)	11,633 (35.74)	1,498 (36.51)	
Moderate	5,911 (17.24)	5,552 (18.02)	359 (9.20)	
Heavy	7,850 (21.61)	7,397 (22.55)	453 (11.94)	
Physical activity, MET-min/week	720.00 (63.00, 2,646.00)	756.00 (105.00, 2,880.00)	240.00 (0.00, 1,440.00)	< 0.001
BMI (%)	28.80 (0.07)	28.65 (0.07)	30.34 (0.16)	< 0.001
Hypertension (%)	16,758 (37.77)	13,362 (34.25)	3,396 (74.05)	< 0.001
Diabetes mellitus (%)	6,740 (12.75)	5,018 (10.52)	1,722 (35.79)	< 0.001
Hyperlipidemia (%)	27,509 (69.58)	23,752 (67.90)	3,757 (86.92)	< 0.001
DI-GM, Mean (SE)	4.58 (0.02)	4.58 (0.02)	4.59 (0.03)	0.34
0–3	10,140 (24.30)	9,111 (24.36)	1,029 (23.68)	0.076
4	10,091 (25.10)	8,975 (25.26)	1,116 (23.50)	
5	9,392 (23.90)	8,262 (23.71)	1,130 (25.80)	
≥6	9,558 (26.70)	8,452 (26.67)	1,106 (27.03)	
Beneficial to gut microbiota Mean (SE)	2.28 (0.02)	2.28 (0.02)	2.22 (0.03)	0.032
Unfavorable to gut microbiota Mean (SE)	2.30 (0.01)	2.29 (0.01)	2.37 (0.02)	0.002

All means and SEs for continuous variables and percentages for categorical variables were weighted.

NHANES, National Health and Nutrition Examination Survey; CVD, cardiovascular disease; PIR, Poverty–income ratio; MET, metabolic equivalent; BMI, body mass index; SE, standard error; DI-GM, dietary index for gut microbiota.

### Association between DI-GM and cardiovascular diseases

Table 2 shows no significant association between DI-GM and CVD in the unadjusted model (OR = 1.00, 95% CI: 0.98–1.03, *P* = 0.768). However, in the fully adjusted model (Model III, adjusted for age, gender, race/ethnicity, education, marital status, PIR, smoking, drinking, physical activity, hypertension, diabetes, hyperlipidemia, and energy intake), each one-point rise in DI-GM was linked to a 5% decrease in CVD prevalence (OR = 0.95, 95% CI: 0.92–0.98). For categorical analysis, DI-GM was grouped into four categories: 0–3, 4, 5, and ≥6, with the 0–3 group serving as the reference, participants with DI-GM ≥ 6 showed a significant negative association with CVD prevalence (OR = 0.82, 95% CI = 0.71–0.94). Furthermore, a higher score for beneficial gut microbiota components was linked to a significant decrease in CVD prevalence (OR = 0.93, 95% CI = 0.89–0.97), while no significant link was observed between unfavorable gut microbiota components and CVD.

**Table 2:** Association between DIGM index and CVD in NHANES 1999–2018.

**Variables**	**Crude model**		**Model I**		**Model II**		**Model III**	
	**OR (95% CI)**	***P*** **-value**	**OR (95% CI)**	***P*** **-value**	**OR (95% CI)**	***P*** **-value**	**OR (95% CI)**	***P*** **-value**
DI-GM	1.00 (0.98, 1.03)	0.768	0.89 (0.86, 0.91)	< 0.001	0.93 (0.90, 0.96)	< 0.001	0.95 (0.92, 0.98)	0.003
**DI-GM group**								
0–3	Reference		Reference		Reference		Reference	
4	0.96 (0.86, 1.07)	0.440	0.77 (0.68, 0.87)	< 0.001	0.81 (0.72, 0.92)	0.001	0.83 (0.73, 0.95)	0.006
5	1.12 (1.00, 1.26)	0.058	0.78 (0.68, 0.90)	< 0.001	0.88 (0.76, 1.01)	0.069	0.91 (0.78, 1.05)	0.182
≥6	1.04 (0.92, 1.18)	0.512	0.61 (0.54, 0.70)	< 0.001	0.75 (0.66, 0.85)	< 0.001	0.82 (0.71, 0.94)	0.004
*P* for trend		0.156		< 0.001		< 0.001		0.021
Beneficial to gut microbiota	0.96 (0.92, 0.99)	0.021	0.84 (0.81, 0.88)	< 0.001	0.91 (0.87, 0.94)	< 0.001	0.93 (0.89, 0.97)	< 0.001
Unfavorable to gut microbiota	1.08 (1.03, 1.13)	0.002	0.97 (0.93, 1.02)	0.282	0.98 (0.93, 1.03)	0.374	0.98 (0.93, 1.04)	0.590

Crude model: no other covariates were adjusted. Model I: Adjust for age, gender. Model II: Adjust for age, gender, race, education, marital status, PIR. Model III: Adjust for age, gender, race, education, marital status, PIR, smoke, drinking status, physical activity, hypertension, diabetes mellitus, hyperlipidemia, energy.

DIGM, dietary index for gut microbiota; CVD, cardiovascular disease; NHANES, National Health and Nutrition Examination Survey; OR, odds ratio; CI, confidence interval; Ref, reference; PIR, Poverty–income ratio.

**Figure 2:**
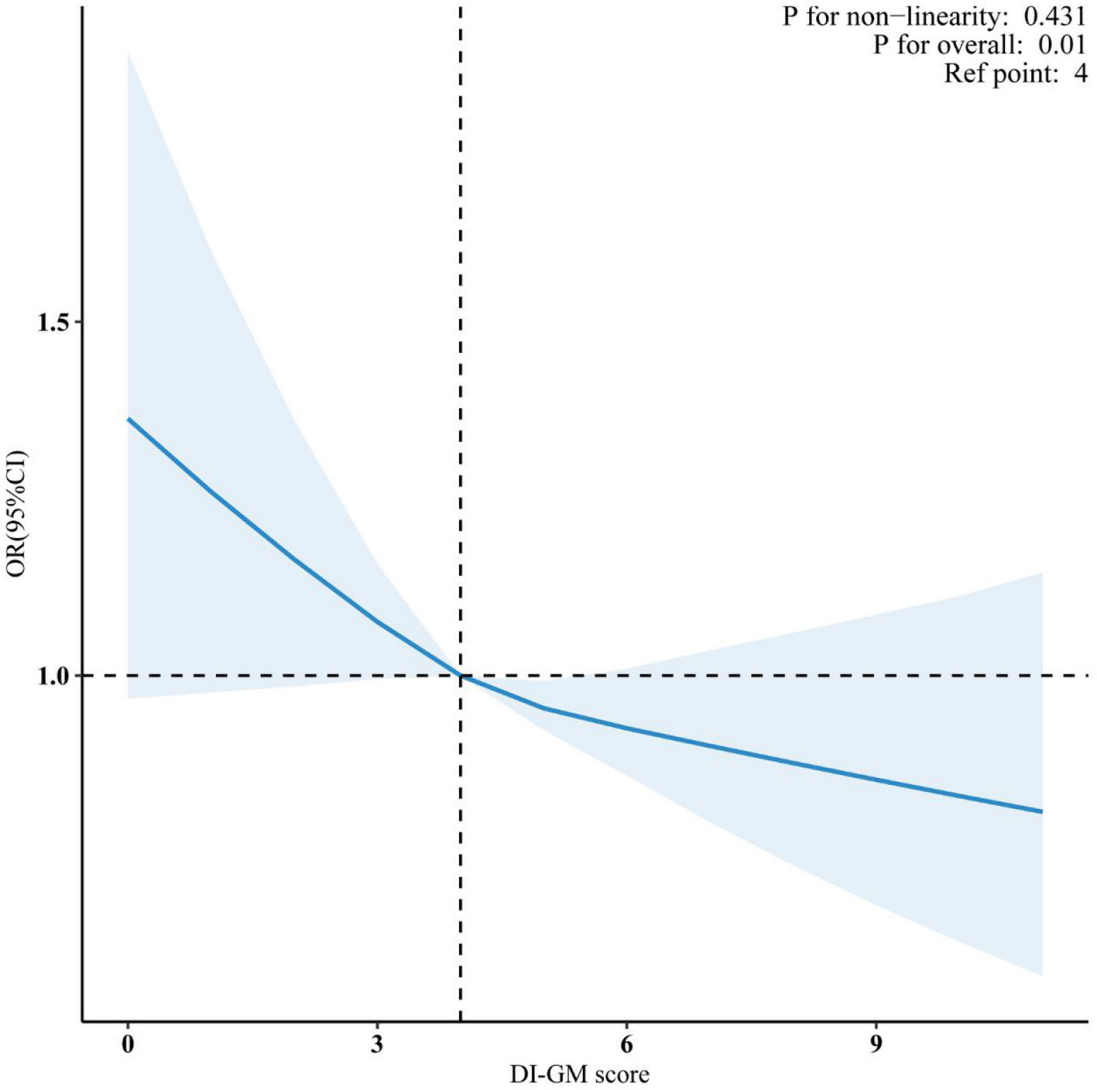
Association between DI-GM and CVD in NHANES 1999–2018 participants. Data were fitted by a survey-weighted multivariable logistic regression model based on restricted cubic splines. Solid and dashed lines represent the predicted value and 95% confidence intervals. They were adjusted for age, gender, race, education, marital status, PIR, smoke, drinking status, physical activity, hypertension, diabetes mellitus and hyperlipidemia. DI-GM, dietary index for gut microbiota; CVD, cardiovascular disease; PIR, poverty income ratio; OR, odds ratio; CI, confidence interval.

Meanwhile, the restricted cubic spline (RCS) analysis showed a linear relationship between DI-GM and CVD (*P* for non-linearity = 0.431; Figure 2). Additionally, both the beneficial (*P* for non-linearity = 0.235) and unfavorable (*P* for non-linearity = 0.694) dietary components for gut microbiota showed a linear association with CVD (Supplementary Figures S1, S2).

### Subgroup analysis of DI-GM and cardiovascular diseases

**Figure 3:**
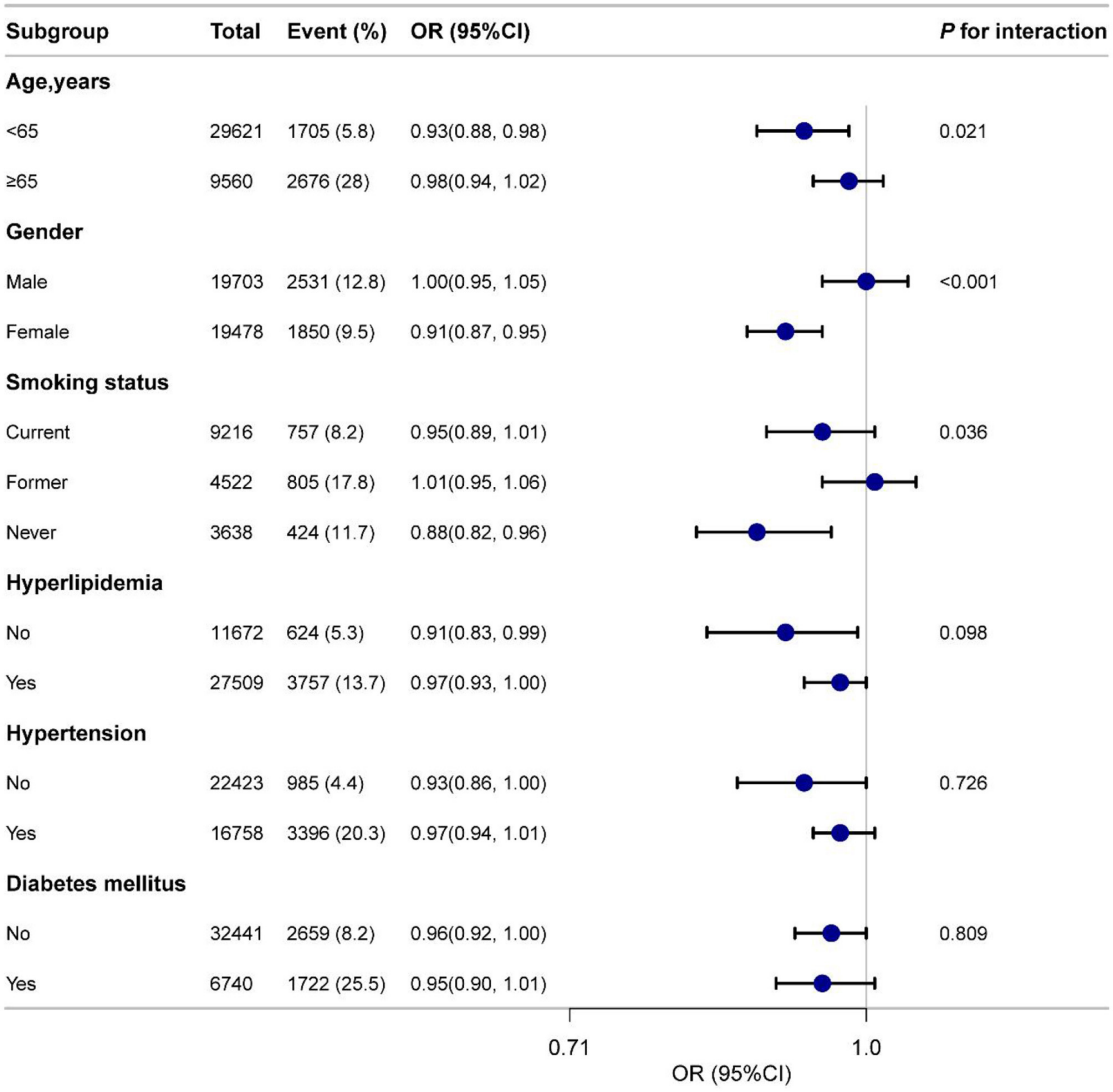
Subgroup analyses of the association between DI-GM and CVD among participants. Adjusted for age, gender, race, education, marital status, PIR, smoke, drinking status, physical activity, hypertension, diabetes mellitus and hyperlipidemia. DI-GM, dietary index for gut microbiota; CVD, cardiovascular disease; PIR, poverty income ratio; OR, odds ratio; CI, confidence interval.

Figure 3 presents the subgroup analyses by age, gender, smoking status, hypertension, diabetes, and hyperlipidemia. Significant interactions were observed for gender (*P* for interaction < 0.001) and age (*P* for interaction = 0.021), but no significant interactions were found for the other subgroups. Specifically, the OR for males was 1.00 (95% CI: 0.95–1.05), indicating no significant association, whereas the OR for females was 0.91 (95% CI: 0.87–0.95), suggesting a significant inverse relationship. These results highlight that gender and age may influence the link between DI-GM and CVD. Overall, the subgroup analyses demonstrated a consistent negative association between DI-GM and CVD across most groups, supporting the generalizability and robustness of the DI-GM across diverse populations.

### Sensitivity analysis

After multiple imputation, the association between DI-GM and CVD remained significant (Model III: OR = 0.97, 95% CI = 0.95–0.99), with DI-GM ≥ 6 also showing a significant relationship (Model III: OR = 0.87, 95% CI = 0.79–0.95), as detailed in Supplementary Table S2. Furthermore, excluding participants with extreme energy intake resulted in a final sample of 38,302, and the association between DI-GM and CVD persisted (Supplementary Table S3).

### Mediation analysis

**Figure 4:**
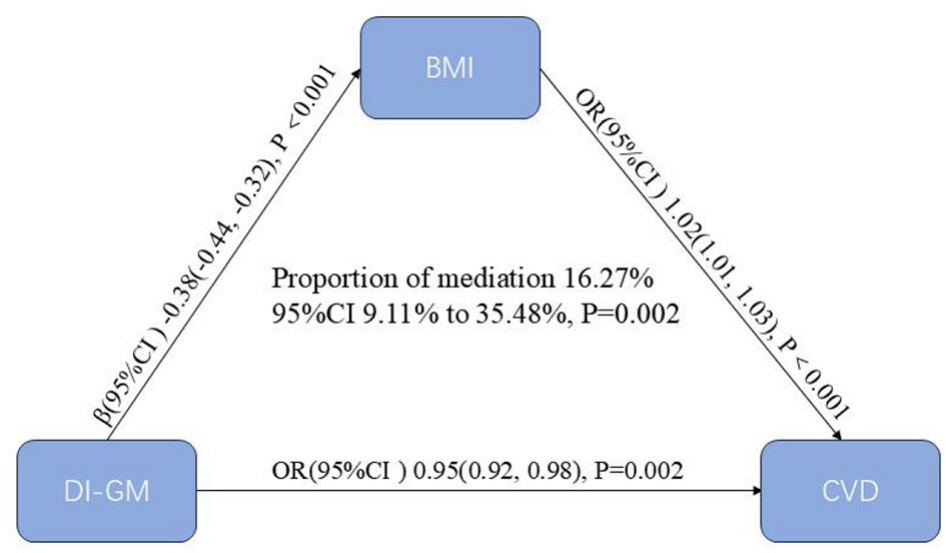
Mediation analysis of BMI in the association between DI-GM and CVD. The models were adjusted for age, gender, race, education, marital status, PIR, smoke, drinking status, physical activity, hypertension, diabetes mellitus and hyperlipidemia. BMI, body mass index; DI-GM, dietary index for gut microbiota; CVD, cardiovascular disease; PIR, poverty income ratio; CI, confidence interval.

Figure 4 illustrates the results of the mediation analysis evaluating BMI as a mediator in the association between DI-GM and CVD. Higher DI-GM scores were associated with lower BMI (β = −0.38, 95% CI: −0.44 to −0.32, *P* < 0.001), indicating an inverse relationship. In turn, higher BMI was positively associated with increased odds of CVD (OR = 1.02, 95% CI = 1.01–1.03, *P* < 0.001). BMI explained 16.27% (95% CI = 9.11–35.48%, *P* = 0.002) of the relationship between DI-GM and CVD, suggesting that part of the protective association between DI-GM and CVD may be explained through its influence on BMI.

## Discussion

Our study found that higher DI-GM scores were linked to a reduced prevalence of CVD, particularly after adjusting for confounders. A one-point increase in DI-GM correlated with a 5% reduction in CVD risk, and participants with DI-GM ≥6 had a significantly lower CVD prevalence. Beneficial dietary components were inversely related to CVD, while BMI partially mediated this association. Subgroup analysis revealed stronger associations in females and variations by age. These results highlight the potential of DI-GM as a dietary marker for CVD prevention, with BMI acting as a mediator.

CVD remains to be a major cause of global morbidity and mortality, with recognized risk factors including hypertension, dyslipidemia, smoking, and physical inactivity (17–20). Despite advancements in prevention and treatment, these factors alone do not fully explain the increasing incidence of CVD, prompting growing interest in diet and gut microbiota. Recent studies emphasize that dietary patterns, particularly those rich in fruits, whole grains, vegetables and healthy fats, can substantially lower the risk of CVD by improving metabolic health and modulating gut microbiota composition (21–24). The gut microbiota, in turn, has been shown to play a crucial role in cardiovascular health (11), with lower microbial diversity linked to conditions such as obesity, diabetes, and coronary artery disease (25–28).

Most existing research has focused either on individual nutrients or microbiota composition in relation to CVD. For instance, the Dietary Approaches to Stop Hypertension (DASH) diet, which emphasizes high fruit, vegetable, and low-fat dairy intake, has been associated with reduced CVD risk in multiple cohorts (29). Other studies have explored the role of specific microbial metabolites, such as trimethylamine-N-oxide (TMAO), in promoting atherosclerosis and thrombosis (30, 31). However, few studies have integrated dietary patterns with microbiota diversity to assess their combined impact on CVD risk.

The DI-GM is a novel dietary index that captures the influence of diet on gut microbiota, a key factor in overall health. Emerging evidence suggests that DI-GM is not only associated with gastrointestinal conditions like constipation but also with broader health outcomes, including metabolic disorders, depression, stroke and increased diabetes risk (13–15, 32, 33). By identifying dietary factors that shape gut microbial diversity, DI-GM provides insights into how specific eating habits may influence health and can serve as a predictive tool for stratifying risk, especially for diseases like CVD. For example, high-fat diets reduce the abundance of beneficial Bacteroidetes while increasing Firmicutes, disrupting energy metabolism (34). In contrast, fiber-rich foods like whole grains, legumes, and fruits support beneficial bacteria such as Bifidobacteria and Lactobacilli, which produce short-chain fatty acids and improve insulin sensitivity (35).

While several dietary indices, such as the Healthy Eating Index (HEI), Mediterranean Diet Score (MDS), and DASH diet, have been developed to assess overall diet quality (23, 29, 36), they do not specifically address the relationship between diet and gut microbiota. Furthermore, their correlations with microbiota diversity and richness remain inconsistent. Recent studies further support the relevance of diet–microbiota interactions to metabolic health. For example, De Matteis et al. (37) reported that lower adherence to a microbiota-supportive Mediterranean diet was associated with increased visceral adiposity and adverse anthropometric indicators. In addition, another study found that low adherence to such dietary patterns was linked to a higher risk of gastrointestinal cancers (38), highlighting the broader implications of microbiota-targeted diets beyond cardiovascular disease. These findings reinforce the value of DI-GM as a focused, microbiota-sensitive index that may help explain dietary contributions to a range of chronic disease outcomes. DI-GM, therefore, offers a more targeted approach by linking diet to microbiota diversity and CVD risk.

BMI appears to play a partial mediating role in the relationship between DI-GM and CVD. Our findings suggest that the beneficial effects of DI-GM on cardiovascular health may be partly attributed to its influence on weight management, as a healthier diet may contribute to lower BMI, which in turn reduces CVD risk. However, it is important to note that BMI explains only part of the relationship, implying that DI-GM may exert direct effects on CVD through other mechanisms, such as inflammation or metabolic regulation, beyond weight management alone.

The relationship between DI-GM and CVD involves multiple mechanisms, primarily through the “gut-heart axis.” Diets with high DI-GM, abundant in fiber and plant-based foods, encourage the growth of beneficial bacteria that produce short-chain fatty acids (SCFAs) like butyrate, known for their anti-inflammatory properties. By reducing systemic inflammation, SCFAs help mitigate the development of atherosclerosis and other cardiovascular conditions (39). Moreover, DI-GM diets improve metabolic health by enhancing glucose metabolism, regulating lipid profiles, and lowering blood pressure (40, 41), all of which are crucial for cardiovascular health. The gut microbiota also influences obesity, a key CVD risk factor, by affecting energy metabolism and fat storage, potentially reducing BMI and overall CVD risk. In addition to SCFAs, other microbiota-derived metabolites such as TMAO, bile acids, and aromatic amino acids play critical roles in cardiovascular regulation. TMAO, for example, promotes vascular inflammation and atherosclerotic plaque formation, while altered BA signaling can affect lipid metabolism and blood pressure regulation. These metabolites act through pathways involving TLR4-NLRP3, FXR, and GPCRs, linking diet-induced microbiota shifts to host cardiovascular outcomes (42). Future research should aim to elucidate these mechanistic pathways using prospective cohort designs or randomized controlled dietary trials.

Gender differences in the effects of DI-GM on CVD risk were also observed in our subgroup analysis. Specifically, DI-GM appeared to have a more pronounced protective effect against CVD in women compared to men. This difference may be due to hormonal variations, differences in dietary habits, and gender-specific differences in gut microbiota composition. Estrogen, for example, has been shown to influence the gut microbiome (43) and may enhance the beneficial effects of a high DI-GM diet. Furthermore, women may be more sensitive to dietary patterns due to hormonal fluctuations that impact metabolism and inflammatory responses (44).

This study has several strengths. To our knowledge, it is the first to explore the association between DI-GM, a dietary quality index linked to gut microbiota diversity, and CVD, while also examining the mediating role of BMI. We utilized nationally representative data from the NHANES, which employs rigorous quality control and a complex multistage sampling design, thereby enhancing the generalizability of our findings to the U.S. adult population. The large sample size and inclusion of diverse demographic subgroups improve statistical power and allow for subgroup and sensitivity analyses. Furthermore, multiple imputation for missing data and robust adjustment for confounders strengthen the internal validity of our results. However, several limitations should be acknowledged. First, its cross-sectional design prevents determination of causal relationships between DI-GM and CVD. Longitudinal cohort studies or randomized trials are needed to confirm these associations. Second, confounding effects from unmeasured or unknown variables may still exist, despite efforts to control for confounders. Third, while DI-GM was derived from 14 foods, NHANES data did not include specific tea types, limiting the inclusion of related dietary parameters. Lastly, the use of self-reported 24-h dietary records and questionnaires to assess DI-GM and CVD may introduce recall bias. Nonetheless, the consistency across sensitivity analyses supports the robustness of the observed associations.

## Conclusions

In this study, higher DI-GM scores were significantly associated with lower prevalence of CVD, and this association was partially mediated by lower BMI. These findings suggest that dietary patterns linked to gut microbiota may play a role in CVD risk. Further longitudinal or interventional studies are needed to validate these associations and explore potential mechanisms.

## Data Availability

Publicly available datasets were analyzed in this study. These survey data are free and publicly available, and can be downloaded directly from the NHANES website (http://www.cdc.gov/nchs/nhanes.htm) by users and researchers worldwide.
