# Supplementary material for: Association of dietary index for gut microbiota and cardiovascular diseases in American adults: evidence from National Health and Nutrition Examination Survey 1999–2018
Source: Front Nutr. 2025 Jul 2;12:1604891. doi: 10.3389/fnut.2025.1604891 (PMC12263386; doi:10.3389/fnut.2025.1604891)
Supplement: Supplementary file 1 [file Data_Sheet_1.docx]

**Supplementary Materials Files**

To: Association of dietary index for gut microbiota and cardiovascular diseases in American Adults：Evidence from National Health and Nutrition Examination Survey 1999-2018

By Hong Luo, Wujie Xia, Liya Pan

**Supplementary Table:**

**Table S1. Components and scoring criteria of DI-GM in NHANES.**

**Table S2 Association between DIGM index and CVD in participants after multiple imputation**

**Table S3. Association between DIGM index and CVD in participants with extreme energy intake was not included**

**Supplementary Figure:**

**Figure S1. Association between beneficial to gut microbiota and CVD in NHANES 1999–2018 participants.**

**Figure S2. Association between unfavorable to gut microbiota and CVD in NHANES 1999–2018 participants.**

**Table S1. Components and scoring criteria of DI-GM in NHANES.**

| **Components of DI-GM** | **Food items included in NHANES** | **Scoring criteria** |
| --- | --- | --- |
| **Beneficial to gut microbiota** | Avocados | Score 1 - Consumption≥sex-specific median  Score 0 - Otherwise |
|  | Broccoli |  |
|  | Chickpeas |  |
|  | Coffee |  |
|  | Cranberries |  |
|  | Fermented dairy (including yogurt, cheese, kefir, sour cream, buttermilk) |  |
|  | Fiber |  |
|  | Soybean (including Soy milk, Tofu) |  |
|  | Whole grains |  |
| **Unfavorable to gut microbiota** | Refined grains | Score 0 - Consumption≥sex-specific median  Score 1 - Otherwise |
|  | Processed meat |  |
|  | Red meat |  |
|  | High-fat diet (% energy) | Score 0 - Consumption≥40%  Score 1 - Otherwise |

DI-GM, dietary index for gut microbiota; NHANES, National Health and Nutrition Examination Survey.

**Table S2** Association between DIGM index and CVD in participants after multiple imputation

| Variables |  | Crude model | | | | Model I | | | | Model II | | | | Model III | | |
| --- | --- | --- | --- | --- | --- | --- | --- | --- | --- | --- | --- | --- | --- | --- | --- | --- |
|  |  | OR (95%CI) | | *P*-value | | OR (95%CI) | | *P*-value | | OR (95%CI) | | *P*-value | | OR (95%CI) | | *P*-value |
| DI-GM |  | 1.03(1.01,1.05) | | 0.001 | | 0.91(0.89,0.93) | | <0.001 | | 0.95(0.93,0.97) | | <0.001 | | 0.97(0.95,0.99) | | 0.002 |
| DI-GM group | | |  | |  | |  | |  | |  | |  | |  | |
| 0-3 |  | Reference | |  | | Reference | |  | | Reference | |  | | Reference | |  |
| 4 |  | 1.07(0.99,1.16) | | 0.083 | | 0.90(0.83,0.98) | | 0.019 | | 0.94(0.86,1.03) | | 0.188 | | 0.97(0.88,1.06) | | 0.439 |
| 5 |  | 1.22(1.13,1.32) | | <0.001 | | 0.88(0.81,0.96) | | 0.004 | | 0.97(0.89,1.06) | | 0.463 | | 1.00(0.92,1.10) | | 0.976 |
| ≥6 |  | 1.16(1.07,1.26) | | <0.001 | | 0.70(0.64,0.76) | | <0.001 | | 0.81(0.74,0.89) | | <0.001 | | 0.87(0.79,0.95) | | 0.002 |
| P for trend |  |  | | <0.001 | |  | | <0.001 | |  | | <0.001 | |  | | 0.007 |
| Beneficial to gut microbiota |  | 0.96(0.94,0.98) | | 0.001 | | 0.87(0.85,0.89) | | <0.001 | | 0.92(0.89,0.94) | | <0.001 | | 0.94(0.91,0.97) | | <0.001 |
| Unfavorable to gut microbiota |  | 1.14(1.11,1.17) | | <0.001 | | 0.99(0.97,1.03) | | 0.733 | | 1.00(0.97,1.03) | | 0.900 | | 1.00 (0.96, 1.03) | | 0.926 |

Crude model: no other covariates were adjusted.

Model I: Adjust for age, gender.

Model II: Adjust for age, gender, race, education, marital status, PIR.

Model III: Adjust for age, gender, race, education, marital status, PIR, smoke, drinking status, physical activity, hypertension, diabetes mellitus, hyperlipidemia, energy.

DIGM, dietary index for gut microbiota; CVD, cardiovascular disease; OR, Odds Ratio; CI, Confidence Interval; Ref, reference; PIR, Poverty–income ratio.

**Table S3.** Association between DIGM index and CVD in participants with extreme energy intake was not included

| Variables |  | Crude model | | | | Model I | | | | Model II | | | | Model III | | |
| --- | --- | --- | --- | --- | --- | --- | --- | --- | --- | --- | --- | --- | --- | --- | --- | --- |
|  |  | OR (95%CI) | | *P*-value | | OR (95%CI) | | *P*-value | | OR (95%CI) | | *P*-value | | OR (95%CI) | | *P*-value |
| DI-GM |  | 1.01(0.98,1.03) | | 0.717 | | 0.89(0.86,0.92) | | <0.001 | | 0.93(0.90,0.96) | | <0.001 | | 0.95(0.92,0.98) | | 0.005 |
| DI-GM group | | |  | |  | |  | |  | |  | |  | |  | |
| 0-3 |  | Reference | |  | | Reference | |  | | Reference | |  | | Reference | |  |
| 4 |  | 0.96(0.85,1.08) | | 0.490 | | 0.77(0.68,0.88) | | <0.001 | | 0.81(0.71,0.93) | | 0.002 | | 0.83(0.72,0.95) | | 0.007 |
| 5 |  | 1.13(1.00,1.27) | | 0.055 | | 0.79(0.68,0.91) | | <0.001 | | 0.89(0.77,1.03) | | 0.111 | | 0.91(0.79,1.06) | | 0.232 |
| ≥6 |  | 1.05(0.92,1.19) | | 0.446 | | 0.62(0.54,0.71) | | <0.001 | | 0.76(0.66,0.87) | | <0.001 | | 0.82(0.71,0.95) | | 0.007 |
| P for trend |  |  | | 0.129 | |  | | <0.001 | |  | | <0.001 | |  | | 0.033 |
| Beneficial to gut microbiota |  | 0.97(0.93,1.00) | | 0.049 | | 0.85(0.82,0.88) | | <0.001 | | 0.91(0.88,0.95) | | <0.001 | | 0.94(0.90,0.98) | | 0.005 |
| Unfavorable to gut microbiota |  | 1.07(1.02,1.12) | | 0.008 | | 0.97(0.92,1.02) | | 0.207 | | 0.97(0.92,1.02) | | 0.276 | | 0.97 (0.92, 1.03) | | 0.272 |

Crude model: no other covariates were adjusted.

Model I: Adjust for age, gender.

Model II: Adjust for age, gender, race, education, marital status, PIR.

Model III: Adjust for age, gender, race, education, marital status, PIR, smoke, drinking status, physical activity, hypertension, diabetes mellitus, hyperlipidemia, energy.

DIGM, dietary index for gut microbiota; CVD, cardiovascular disease; OR, Odds Ratio; CI, Confidence Interval; Ref, reference; PIR, Poverty–income ratio.


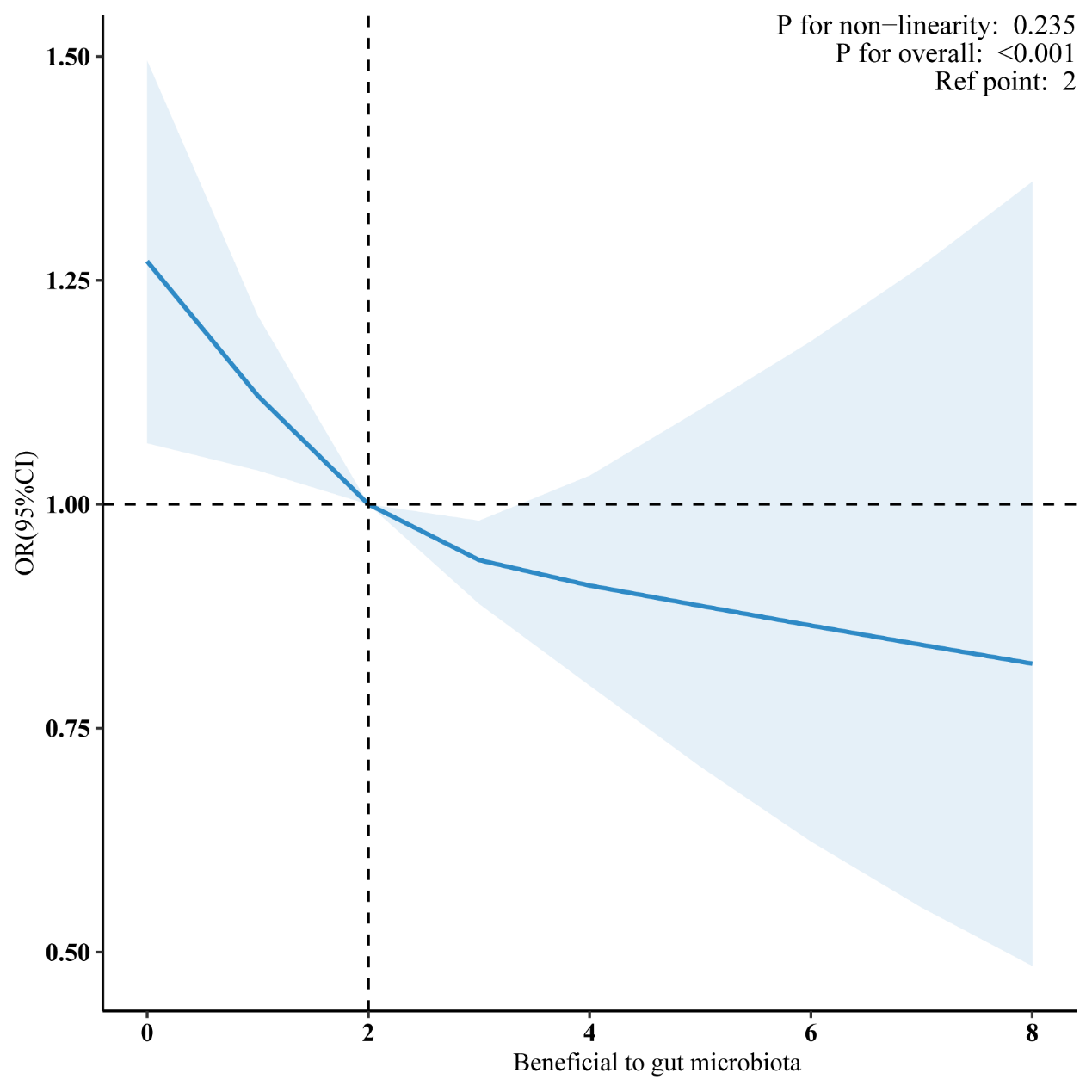


**Figure S1.** Association between beneficial to gut microbiota and CVD in NHANES 1999–2018 participants. Data were ﬁtted by a survey-weighted multivariable logistic regression model based on restricted cubic splines. Solid and dashed lines represent the predicted value and 95% confidence intervals. They were adjusted for age, gender, race, education, marital status, PIR, smoke, drinking status, physical activity, hypertension, diabetes mellitus and hyperlipidemia. DI-GM, dietary index for gut microbiota; CVD, cardiovascular disease; PIR, poverty income ratio; OR, Odds Ratio; CI, Confidence Interval.


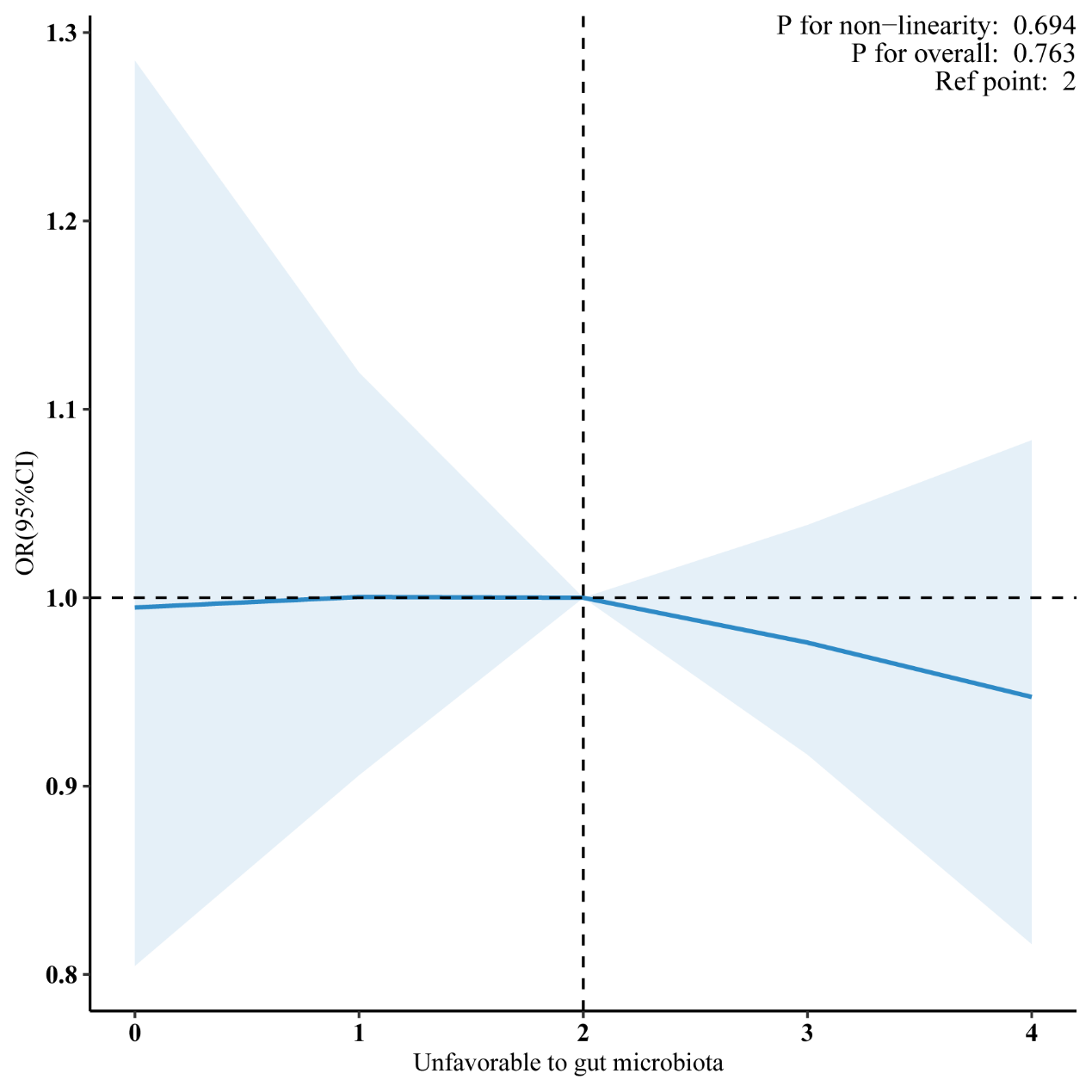


**Figure S2.** Association between unfavorable to gut microbiota and CVD in NHANES 1999–2018 participants. Data were ﬁtted by a survey-weighted multivariable logistic regression model based on restricted cubic splines. Solid and dashed lines represent the predicted value and 95% confidence intervals. They were adjusted for age, gender, race, education, marital status, PIR, smoke, drinking status, physical activity, hypertension, diabetes mellitus and hyperlipidemia. DI-GM, dietary index for gut microbiota; CVD, cardiovascular disease; PIR, poverty income ratio; OR, Odds Ratio; CI, Confidence Interval.
